# Complete Genome Sequence of Halophilic Deep-Sea Bacterium Halomonas axialensis Strain Althf1

**DOI:** 10.1128/MRA.00839-19

**Published:** 2019-08-01

**Authors:** Megumi Tsurumaki, Senka Deno, Josephine Galipon, Kazuharu Arakawa

**Affiliations:** a Institute for Advanced Biosciences, Keio University, Tsuruoka, Japan; b Systems Biology Program, Graduate School of Media and Governance, Keio University, Fujisawa, Japan; c Faculty of Environment and Information Studies, Keio University, Fujisawa, Japan; University of Southern California

## Abstract

Halomonas axialensis is a halophilic bacterial species discovered near a deep-sea hydrothermal vent. Here, we report the first single closed genome sequence of the original strain, Halomonas axialensis strain Althf1. The genome was assembled by Nanopore sequencing and consisted of a single chromosome of 3.6 Mbp with 56.8% G+C content.

## ANNOUNCEMENT

Halomonas axialensis is a deep-sea halophilic bacterium most closely related to Halomonas aquamarina and Halomonas meridian (1). It was first isolated by Kaye et al. from low-temperature hydrothermal fluid (Cloud vent) at 1,530 m depth on the Axial Seamount on the Juan de Fuca Ridge in the Northeast Pacific Ocean (1, 2). Strain Althf1 was the first *H. axialensis* strain to be described. Also, a draft genome sequence containing 122 contigs (*N*_50_ value, 70,749 bp) for *H. axialensis* strain ACH-L-8, isolated from the South China Sea at 2,000 m depth, has been announced (3). Here, we report the first complete genome sequence (*N*_50_ value, 3,619,799 bp) of *H. axialensis* strain Althf1, obtained by Nanopore sequencing, to better understand deep-sea *Halomonas* biology.

*H. axialensis* strain Althf1 (ATCC BAA-802 [2], obtained directly from J. Z. Kaye) was cultured using ATCC medium (1097 *Halomonas* medium, with Casamino Acids replaced by high-molecular-weight casein). Total genomic DNA was extracted with a Genomic-tip 20/G (Qiagen), following the manufacturer’s protocol. Sequencing libraries were prepared using the Rapid Barcoding Sequencing protocol (SQK-RBK004) provided by Oxford Nanopore Technologies. Long-read sequencing was performed using the GridION platform (Oxford Nanopore Technologies) with the FLO-MIN106 flow cell, and the quality of sequencing was monitored from the MinKNOW interface (Oxford Nanopore Technologies). Sequences were base called and demultiplexed using the Albacore software version 2.2.6 (Oxford Nanopore Technologies), and a total of 95,277 reads were obtained, with an average length of 5,471 bp. Reads with a length of 10,000 bp or more were retained for the assembly process, resulting in around 100× coverage. The resulting sequences were assembled with Canu version 1.7.1 (4), and the assembled single contig was manually circularized by eliminating an overlapping end. Assembly errors were corrected using nanopolish version 0.10.2. Assembly completeness was assessed by BUSCO v1 (5) on the gVolante server (6) and CheckM (7). Annotation was performed using DFAST v1.1.0 from the DDBJ (8). Two rounds of nanopolish error correction improved and saturated BUSCO completeness from 80% to 95% and CheckM completeness from 79.57% to 88.19%. The CheckM contamination level was 0.47%. The remaining indel rate was calculated by mapping the ACH-L-8 genes onto the Althf1 genome with minimap2 (9), and there were 2,959 deletions (0.11%) and 6,502 insertions (0.24%) out of the mapped 2,647,649-bp regions. Since the average length of a protein-coding sequence (CDS) is 933 bp, a CDS is likely to contain 1 deletion or 2 insertions, resulting in fragmented gene predictions. The remaining base mismatch error is difficult to assess, since there are variations within the strains, but it is estimated to be 2.40% at maximum (25,721 mismatches in 1,070,325 bp of 1,818 genes fully aligned between the two strains with BLASTN [10]). Note that shorter genes are more likely to be free of indels and are more likely to be aligned at full length. All software was run with default parameters.

The complete genome of this strain is 3,619,799 bp, with a G+C content of 56.8%. DFAST identified 5,503 predicted protein-coding sequences (CDS), 18 rRNA genes, and 62 tRNA genes. The genome of *H. axialensis* strain ACH-L-8 was previously reported to contain 20 aldehyde dehydrogenase (ALDH) genes (3) and assumed to be of interest for the bioremediation of aldehyde-contaminated environments. Our DFAST reanalysis of strain ACH-L-8 confirmed the presence of 17 such ALDH coding sequences, 16 of which were also detected by BLASTN 2.8.1+ (10) in the newly sequenced *H. axialensis* strain Althf1. Both strains harbored genes involved in resistance to mercury and arsenic.

This is the first report of a complete genome sequence of *H. axialensis*, as well as the first report for the strain that initially enabled the identification of this species (2). This assembly will facilitate genome-wide comparison studies with a focus on the ecology of bacteria living in deep-sea hydrothermal environments.

### Data availability.

The chromosome and plasmid sequences reported here were deposited in the DDBJ under the accession number AP019517 and in the Sequence Read Archive (SRA) under BioProject accession number PRJNA521630.
